# Neural dynamics of cue reliability in perceptual decisions

**DOI:** 10.1167/jov.20.8.23

**Published:** 2020-08-25

**Authors:** Giovanni Mancuso, Gijs Plomp

**Affiliations:** Perceptual Networks Group, Department of Psychology, University of Fribourg, Fribourg, Switzerland

**Keywords:** detection/discrimination, shape and contour, 3D surface and shape perception, visual evoked potential, uncertainty

## Abstract

To extract meaningful information from scenes, the visual system must combine local cues that can vary greatly in their degree of reliability. Here, we asked whether cue reliability mostly affects visual or decision-related processes, using visual evoked potentials (VEPs) and a model-based approach to identify when and where stimulus-evoked brain activity reflects cue reliability. Participants performed a shape discrimination task on Gaborized ellipses, while we parametrically and independently, varied the reliability of contour or surface cues. We modeled the expected behavioral performance as a linear function of cue reliability and established at what latencies and electrodes VEP activity reflected behavioral sensitivity to cue reliability. We found that VEPs were linearly related to the individual behavioral predictors at around 400 ms post-stimulus, at electrodes over parietal and lateral temporal cortex. The observed cue reliability effects were similar for variations in contour and surface cues. Notably, effects of cue reliability were absent at earlier latencies where visual shape information is typically reported, and also in data time-locked to the behavioral response, suggesting the effects are not decision-related. These results indicate that reliability of visual cues is reflected in late distributed perceptual processes.

## Introduction

The human visual system operates to support complex perceptual decisions and actions. In this process, local cues can help identify contours and surfaces that are combined into shapes and objects, but the reliability of these local cues varies greatly in natural circumstances. The visual system, therefore, faces the challenge of combining cues of variable reliability but it remains an important open question how cue reliability affects stimulus processing. Perceptual performance is known to benefit from a strategic allocation of perceptual resources, such as proportionally weighing the contribution of sensory elements to the final percept based on their relative precision. Studies of multisensory (Angelaki, Gu, & DeAngelis, 2009; Ernst & Banks, 2002; Fetsch, Pouget, DeAngelis, & Angelaki, 2012) as well as unisensory integration (Machilsen & Wagemans, 2011) show that reliable inputs exert stronger influence on perceptual choices than unreliable ones, and that multiple cues are integrated for perceptual decisions. It remains unclear, however, whether variations in the reliability of cues affect early sensory processes or if they are part of later decisional stages. Although previous functional magnetic resonance imaging (fMRI) work showed that stimulus uncertainty is reflected in primary visual areas (van Bergen, Ji Ma, Pratte, & Jehee, 2015; Vilares, Howard, Fernandes, Gottfried, & Kording, 2012), fMRI does not have the necessary temporal resolution to establish temporal precedence.

The dynamics of visual processes can be investigated with electroencephalography (EEG), which allows separating visual and later decisional processes in time. The stimulus evoked electrical brain activity, or visual evoked potential (VEP), typically shows a cascade of highly dynamic processes that first reflect simple stimulus properties at around 100 ms after stimulus onset, then shape and object-specific computations at mid-latencies, and finally decision-related activity at latencies beyond 250 ms (Ales, Appelbaum, Cottereau, & Norcia, 2013; Cichy, Pantazis, & Oliva, 2014; Heekeren, Marrett, & Ungerleider, 2008; Picton, 1992; Plomp, Michel, & Herzog, 2010). Here, we combined EEG with a model-based analyses to determine at what latencies and electrode locations VEPs parametrically reflect visual cue reliability.

Two important cues for shape processing are contours and surfaces (Julesz, 1981; Lamme, 1995; Nothdurft, 1991; Roelfsema, 2006), and their processing dynamics are relatively well understood. Collinear contours and coherent surface textures increase VEP amplitudes from about 100 ms after stimulus onset (Scholte, Jolij, Fahrenfort, & Lamme, 2008; Shpaner, Molholm, Forde, & Foxe, 2013). In a shape detection task with arrays formed of Gabor elements, the presence of aligned contours evokes larger amplitudes of the N1, a component with negative voltage amplitude typically observed at mid latencies (around 170 ms) and a later positive component around after 200 ms (P2) (Machilsen, Novitskiy, Vancleef, & Wagemans, 2011). Detectable contours elicited a larger VEP, especially over occipital electrode sites (Mathes, Trenner, & Fahle, 2006). Because reliable contour and surface cues form shapes and objects (Wagemans, Elder, Kubovy, Palmer, Peterson, Singh, & von der Heydt, 2012), the effects of cue reliability may be expected at latencies where shape-specific responses occur in the EEG. However, the research above typically compared a shape with a non-shape condition, but never parametrically varied cue reliability. In our study, we therefore parametrically varied the reliability of contour and surface cues in a shape discrimination paradigm.

Although parametric effects of cue reliability may be expected from 100 ms onward, the integration of visual information for decisions typically occurs at later stages. Perceptual decisions can be modeled as an evidence accumulation process, the outcome of which determines the appropriate behavioral outputs (Donner, Siegel, Fries, & Engel, 2009; Gold & Shadlen, 2007; Heekeren et al., 2008). In EEG studies, such decision processes are reflected over parietal areas and resemble a classic target-detection component, at 300 ms after stimulus onset (Kelly & O'Connell, 2013; Polanía, Krajbich, Grueschow, & Ruff, 2014; Twomey, Murphy, Kelly, & O'Connell, 2015; Wyart, de Gardelle, Scholl, & Summerfield, 2012). These decision processes have been shown to parametrically reflect stimulus visibility in a categorical discrimination task (Philiastides & Sajda, 2006). The authors rendered low-level cues less reliable for perceptual decisions by scrambling images of faces and cars. The effects of this manipulation were most strongly reflected at latencies after 300 ms, and can be interpreted as an integration process for decisions (Philiastides, Heekeren, & Sajda, 2014).

**Figure 1.**
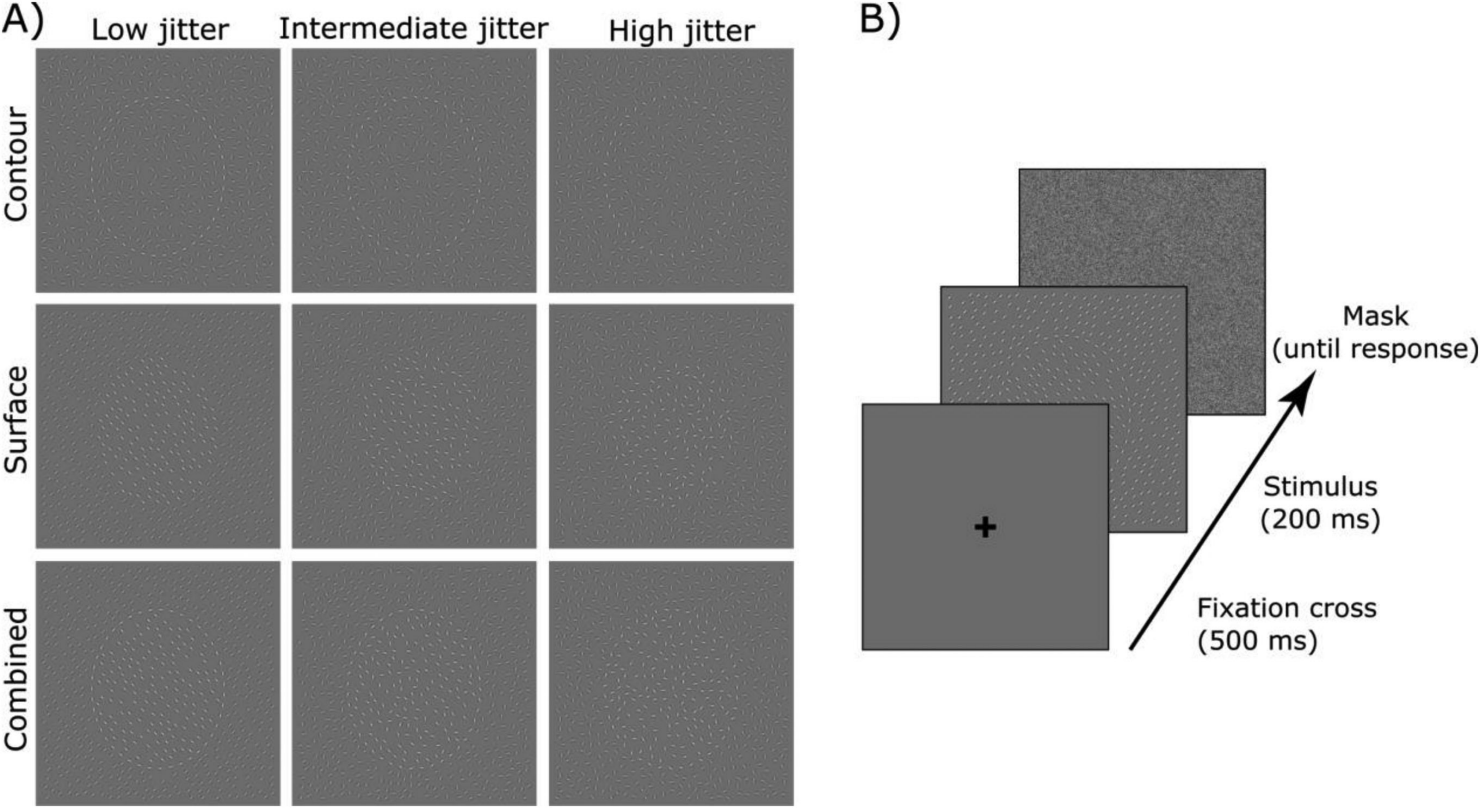
(A) Examples of Gaborized stimulus displays. In the first row, an ellipsoid shape is defined by the collinearity of contour elements – the orientation of each Gabor element is parallel to the tangent of the ellipse outline. In the second row, the shape is defined by isolinearity of the Gabor patches inside the shape. In the last row, both contour and surface elements help define the shape. In the first column, contour and surface cue reliability is maximal: Gabor elements are oriented tangentially to the outline of the ellipse on the Contour and/or have the same orientation in the Surface. The next two columns present reduced reliability by increasing levels of jitter. For illustration purposes, the contrast of contour and surface elements has been increased; in actual stimulus displays all Gabor patches were of equal contrast. (B) Shows the trial sequence for the behavioral session, in the EEG session a variable delay was added to the fixation point.

Late effects of stimulus reliability have typically been obtained using complex stimuli, like faces or objects. Instead, here, we studied well-controlled simplified shapes and prompted figure-ground segregation by leveraging perceptual grouping of contour and surface cues (Machilsen & Wagemans, 2011). Participants were presented with displays of randomly oriented Gabor elements in which contour and surface cues were coherently manipulated to form ellipsoid shapes (Figure 1). When contour and surface cues are presented together, participants integrate the cues and show improved performance (Hess, Hayes, & Field, 2003; Machilsen & Wagemans, 2011; Loffler, 2008; Peirce, 2015; Wagemans et al., 2012). These Gaborized stimulus displays allow full control over low-level features like local contrast and spatial frequency, while at the same time controlling the amount of task-relevant information contained in the display. During EEG recording, we independently varied the reliability of either the contour or surface cues while the other cue was held at an individually calibrated level of discriminability, established in a prior behavioral session. This manipulation allowed us to identify which EEG electrodes and time-windows parametrically reflected cue reliability by matching linear models of evoked activity to the psychophysically derived sensitivities to cue reliability; thus effectively equating behavioral performance curves with neural activity (Britten, Shadlen, Newsome, & Movshon, 1992; Philiastides & Sajda, 2006).

Our results show that effects of cue reliably occur at relatively long latencies, beyond 360 ms after stimulus onset, over central and lateral-frontal electrodes. Our model-based fitting, together with control analyses, support the notion that effects of cue reliability manifest in late stages of sensory processing, after the early and mid-level sensory processes typically reported for shape processing, but before, and not tightly locked to, the execution of the motor response. This implies that visual cues for decisions and action are not necessarily fully integrated at latencies of visual processes that typically reflect shapes and objects, but can occur at longer latencies.

## Methods

### Participants

A total of 16 participants (11 women), between 19 and 26 years of age (mean 22.2 ± 2.4 SD), took part in a behavioral and an EEG session. Participants were recruited at the University of Fribourg and received course credit for participation. Informed consent was obtained prior to participation. All participants had normal or corrected-to-normal visual acuity (> 1) as established with the Freiburg acuity test (Bach, 2006). All procedures complied with the Declaration of Helsinki and were approved by the institutional ethics board.

### Stimuli and material

Stimulus displays were arrangements of non-overlapping Gabor elements presented on a grey background, covering an area of 1000 × 1000 pixels, corresponding to 16 and 17 degrees of visual angle in the behavioral and EEG sessions respectively. Each Gabor element was defined as the product of an oriented sine-wave luminance grating (spatial frequency of 3 cycles per ∼0.7 degrees of visual angle at 50% Michelson contrast) and a circular Gaussian envelope of 3.8 arc minute standard deviation. Each display contained a Gaborized ellipsoid embedded in a field of background elements. We distinguished three regions in each display: (1) the contour region, comprising all elements on the outline of the ellipse, (2) the surface region, comprising all elements inside the contour region, and (3) the background region, comprising all elements outside of the contour. For each display, a total of 590 Gabor elements were distributed as follows: 43 on the contour, 150 on the surface, and the remaining 397 in the background region. The contour cues provide the most reliable information when they are oriented tangentially to the outline of the ellipse; surface cues are maximally reliable when they all have identical orientation (see Figure 1A).

We created three types of displays. In the Contour Condition, the ellipse was solely defined by the alignment of Gabor elements in the contour region, while the elements in the surface and background region were randomly oriented. Similarly, in the Surface Condition, surface elements inside the ellipse were iso-oriented −45° away from vertical, while contour and background elements were oriented +45° away from vertical. In the Combined Condition, both contour and surface cues were aligned to form an ellipse. In the behavioral session, all three display types were presented, in the EEG session, only Combined displays were used.

We manipulated cue reliability by adding random deviations to the orientation of elements in the contour and surface regions. We call these random deviations jitter. In the behavioral session, the amount of jitter across elements was drawn from a normal distribution with mean zero and standard deviation of 1°, 13.8°, 26.6°, 39.4°, 52.2°, or 65°. In the EEG session, jitter levels were adapted to individual performance as measured during the behavioral session.

To remove local density cues that could contribute to correct discrimination of the ellipsoid shape, we statistically controlled the average Euclidean distance between each display element and its four nearest neighbors. An unpaired *t*-test (alpha 0.1) with correction for unequal variances was used to exclude displays with significant differences in the local Euclidean distance among the contour, surface, and background regions of each display (Demeyer & Machilsen, 2012). Across displays, the center-to-center distance of elements was on average 38 arc minute in the behavioral and 40.9 arc minute in the EEG session, with ellipses spanning 10.2 × 8.50 and 11.2 × 9.3 degrees of visual angle, respectively. The major/minor axis ratio of the ellipse was fixed at 1.2 in both sessions. To make strategies to detect shape boundaries at specific display locations effectively suboptimal, we randomly displaced stimulus center to four possible locations (±0.69 degrees of visual angle along the vertical or horizontal meridian). The displaced locations were fully randomized against jitter levels, contour or surface condition, and ellipse orientation. We used 1000 × 1000 pixel noise masks with each pixel intensity sampled from a uniform distribution.

Stimuli were presented on a Philips 202P7 CRT (1600 × 1200, 85 Hz, behavioral session) and a VIEWPixx/3D (1920 × 1080, 100 Hz, EEG session). Custom made Matlab (The Mathworks, Natick, MA) scripts, the Psychophysics Toolbox 3.8 (Branard & Vision, 1997) and GERT (Demeyer & Machilsen, 2012) supported the creation and presentation of the stimuli.

### Procedure

Each participant first completed a behavioral and then an EEG session, scheduled on separate days. All sessions took place in a darkened room where participants sat with the head comfortably positioned on a chin rest at a viewing distance of 90 cm from the monitor. The behavioral session started after dark adaptation and a practice session with easily discriminable stimuli was run until participants accurately judged 20 trials.

The participants were instructed to discriminate whether the stimulus presented contained a vertical or horizontal ellipse, and to respond as accurately and quickly as possible. Task difficulty was manipulated within blocks with the method of constant stimuli and six levels of jitter added to the contour or surface cues. The behavioral session used a blocked within-subject design with three conditions (Contour, Surface, and Combined). In the Combined condition, contour and surface cues were jittered by the same amount. The analysis of Combined condition allowed us to test the cue integration hypothesis that observers benefit from the simultaneous presence of Contour and Surface cues. We fully randomized block orders across participants. In the EEG session, all stimuli comprised contour and surface information together. The jitter of one cue was varied using a blocked within-subject design with the conditions Contour fixed and Surface fixed, using individually calibrated jitter levels (see Behavioral modeling section). To reduce fatigue, blocks were about 10 minutes each (432 trials) and 2 blocks of each condition were presented using simple alternate randomization of the starting block to counterbalance order effects (A-B-A-B versus B-A-B-A). For each session, blocks comprised 432 trials equally divided across the 6 levels of the covariate jitter thus resulting in 72 repetitions. Each condition was repeated twice resulting in a total of 1728 trials. Experimental sessions lasted between 45 minutes and 1 hour.

Trials started with a fixation cross presented at the center of the screen (see Figure 1B). After 500 ms, the fixation was replaced by the visual stimulus presented for 200 ms. The stimulus was then masked until the button press. Participants indicated the orientation of the ellipsoid (vertical or horizontal), by pressing the key “n” or “m,” respectively. There was no maximum response delay. In the EEG session, four possible inter stimulus intervals were introduced between the response and the next stimulus presentation (i.e. fixed cross duration was 500, 550, 600, or 650 ms).

### Behavioral modeling

Individual behavioral data were modeled with a four-parameter normal cumulative function, fitted using the Palamedes Toolbox (Kingdom & Prins, 2016). Although we used probit regression to derive psychophysical curves, we note that our experimental approach deviates from standard signal detection theory. The experimental task was to discriminate stimulus shape (vertical/horizontal ellipse), but instead of varying the signal along the “judgment” axis (vertical-horizontal), as is typical in signal detection theory, we varied the reliability of the cues that defined stimulus shape (c.f.; Jeon, Lu, & Dosher, 2009). From the fitted functions, two parameters of interest were derived. The first was the inflection point of the curve (midpoint between the upper and lower asymptotes; the higher the better performance), referred to as threshold here, the second was the gradient of the curve at the inflection point (a measure of sensitivity to cue reliability, the steeper the faster performance increases for more reliable cues), referred to as slope. The thus defined thresholds, and slopes were estimated for each participant and then submitted to further group-level inferential statistics; the nuisance parameters (lapse and guess rate) were estimated but not evaluated further (Wichmann & Hill, 2001). We set guess rate as a free parameter to accommodate possible random variations in performance.

**Figure 2.**
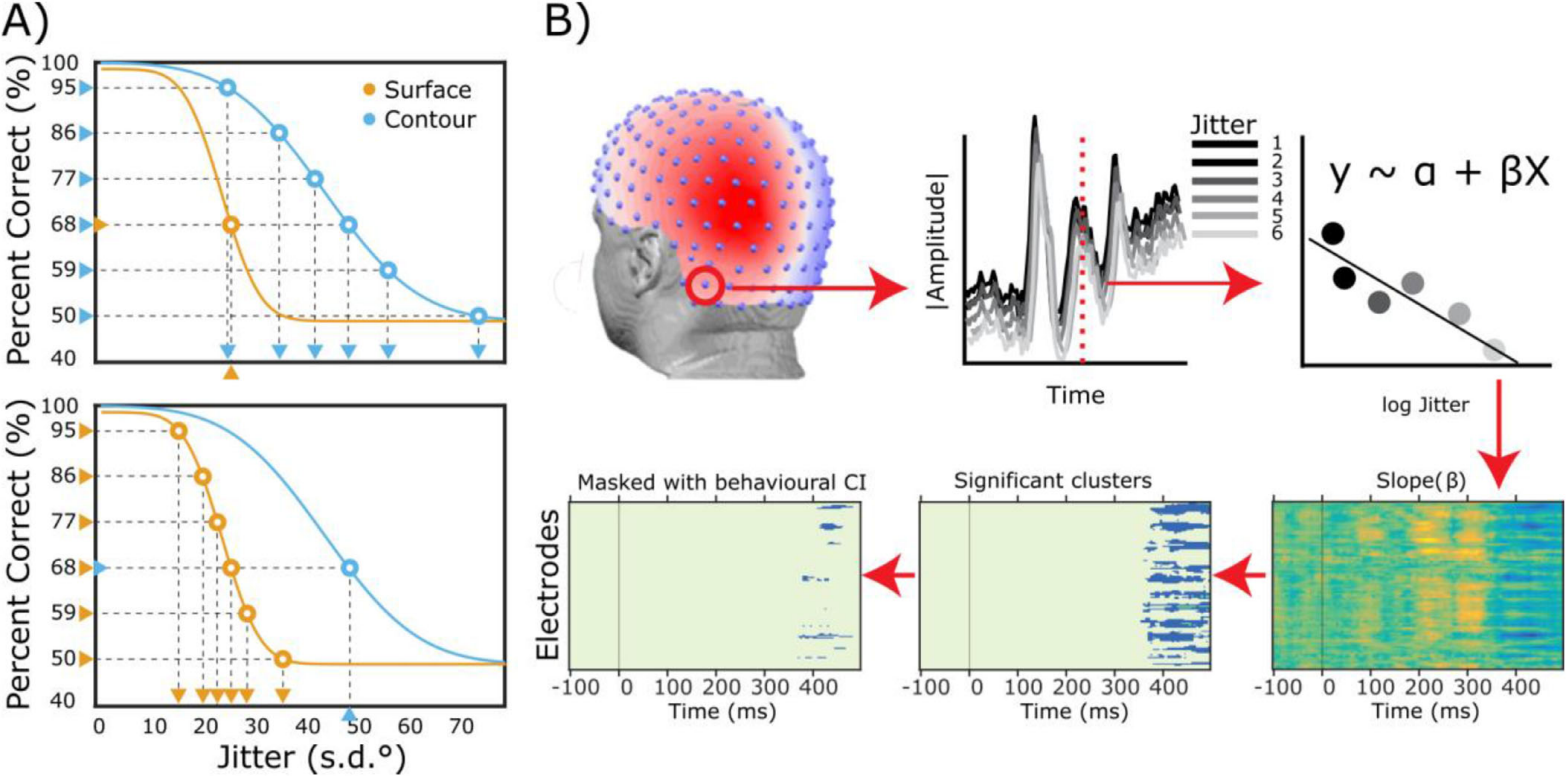
Behavioral and EEG methods. (A) The inverse psychometric function and the selection of individual jitter levels for the EEG session. Each plot shows the performance of a typical subject to surface and contour conditions. The two curves show very different slopes (gradients at inflection point) and thresholds (inflection points), reflecting different sensitivities to contour and surface information. Using the inverse of the fitted probit function, we build combined stimuli that are equally difficult. In the plot on the top, the level of surface jitter is held constant at 68%, while variable levels of jitter for the contour cues are selected (95, 86, 77, 68, 59, and 50%). Likewise, individual values of surface cue jitter were selected when contour cues were fixed (bottom plot). (B) Illustrates the EEG model fitting approach. For each EEG electrode (top-left panel), the rectified amplitude was regressed against the jitter levels (top center and right panels; grand-average data for illustration). The collection of all estimated slopes resulted in a raster plot representation (bottom-right panel), which was then subjected to cluster-based permutation to identify clusters where slope deviated significantly from zero, while controlling for multiple comparison testing (bottom-center panel). Of all the significant clusters, only those that fell within the 95% confidence interval (CI) of behavioral slopes (d′-jitter) were retained for further interpretation (bottom-left panel).

To account for individual differences in performance with contour and surface cues, we created combined stimuli for the EEG session that matched individual sensitivities. This calibration was aimed at controlling the amount of information conveyed by contour and surface cues for the EEG session. In addition, this helps avoid the adoption of veto strategies whereby one cue is systematically disregarded (Landy, Maloney, Johnston, & Young, 1995). Using percentage correct data from the behavioral session we first estimated individual probit function parameters for the Surface and Contour cue conditions. Then, using the inverse of the fitted functions, we generated stimuli with contour and surface cues at fixed levels of performance. Specifically, we fixed the jitter of one cue at 68% of correct responses so that it retained behavioral relevance (Landy et al., 1995), and then varied the jitter of the second cue between 95% and 50% in 6 equidistant steps (95, 86, 77, 68, 59, and 50%). We repeated this procedure for both cues, aiming to create Surface fixed and Contour fixed combined stimuli of equal difficulty (Figure 2A).

Our goal was to determine what electrodes and latencies parametrically reflect cue reliability. One obstacle to fitting the same behavioral psychometric function to neural data concerns the configuration of asymptotes boundaries. Asymptotes, in the behavioral psychometric function, have both a statistical interpretation (bounds of the binary variables, i.e. 100%–50%) and a psychological interpretation (lapse and guess rate for the upper and lower asymptote respectively). In contrast, fitting a four-parameter probit regression model to neural data requires setting the asymptotes boundaries at arbitrary values (micro volts) with the realization that such boundaries have no meaningful interpretation. For these reasons, we re-expressed the behavioral data using a *d'* transformation. Specifically, hits and misses within a single jitter level were expressed as *d’* and then the (logarithm of) *d'* was regressed against the (logarithm of) jitter (see Equation 1 below). Although *d’* is dimensionless and represents the internal strength of a stimulus in standardized units, this linear model incorporates the relationship between sensitivity and jitter - a distinctive feature of a psychometric function. The linear association between *d’* and jitter constituted the starting point of the neurometric fitting as explained in the next section. The linear model can be expressed as follows:

(1) $$log\left(d^{'}\right)∼\beta_{0}+\beta_{1}log\left(jitter\right)+ɛ$$

where β_0_ and β_1_ represent, respectively, the intercept and the slope of the model and ε is normally distributed noise with mean 0 and standard deviation σ(εi.i.d. *N*(0, σ)).

### EEG recording and analysis

EEG data were acquired with a BioSemi Active Two system (Amsterdam, The Netherlands) and 128 active pin-type-electrodes positioned in a headcap that was individually placed such that the cZ electrode was halfway between nasion and inion, and equally far from each ear. Data were recorded at a sampling rate of 1024 Hz. Throughout the recording, good signal quality was guaranteed by keeping the offset between the active electrodes and the CMS-DRL feedback loop, under a standard value of ±20 mV. After each recording session, 3D electrode positions were digitized using an ultrasound motion capture system (Zebris Medical GmbH). EEG pre-processing and analysis were done using EEGLAB (Delorme & Makeig, 2004) and custom made MatLab scripts.

The continuous signal was downsampled to 200 Hz after applying an anti-aliasing filter (cut-off frequency: 160 Hz; transition bandwidth: 80 Hz). Then a high-pass (> 0.1 Hz) non-causal FIR filter was applied. The Cleanline EEGLAB plugin was used to remove 50 Hz line noise (and its harmonics) and the EEG signal was parsed into 2-second epochs centered on stimulus onset. Epochs with non-stereotypical artifacts were identified by qualitative inspection and not further analyzed (on average, 15 ± 5% of trials). Muscular and ocular artifacts were removed using independent component analysis (ICA, EEGLAB, and extended Infomax algorithm; (Delorme & Makeig, 2004). Epochs were re-referenced to the average signal across electrodes. For the analysis of response-locked activity, the same preprocessing was applied to 2-second epochs centered on the response. On average, 22 ± 3% of response-locked epochs were not further analyzed due to artifacts.

To identify at what electrodes and latencies cue reliability are encoded in VEPs, we linearly regressed VEP amplitudes against jitter levels at each electrode and at each time point around stimulus onset (−100 to +500 ms; for response-locked analysis: −1000 to 0 ms). For this analysis, individual EEG signals were *z*-scored across trials and electrodes. We rectified signals before the regression because positive and negative amplitudes of the scalp EEG both signal current flows (activity) in the underlying tissue (Nunez & Srinivasan, 2006). Finally, a linear model was estimated regressing the rectified signal against the six levels of jitter, iterating through electrodes and time points. This iterative procedure was fit separately to data from the Contour and Surface fixed conditions (see Figure 2B).

By modeling VEPs as a function of jitter level, we estimated slope parameters for each participant, electrode, and time point. Positive and negative slope values were submitted to a group-level cluster-based permutation test (Bullmore et al., 1999; Maris & Oostenveld, 2007) to jointly account for spatial and temporal correlations and control for multiple comparisons (two-tailed test, with 5000 permutation and a nominal significance value of *p* < 0.01). We selected all significant clusters whose average slopes fell within the 95% confidence intervals of the predicted behavioral model (*d’-*jitter association). This means that of all significant clusters identified with the cluster-based permutation test, only those that fell within the confidence intervals of the estimated slope in the behavioral regression were retained for further analysis (see Figure 2). The same analysis was applied to stimulus- and response-locked data. We assumed that EEG activity would increase with cue reliability because contours and surfaces typically give rise to more EEG activity than control stimuli (Machilsen et al., 2011; Scholte et al., 2008; Shpaner et al., 2013), and because previous work indicated that neural activity can follow psychometric curves (Britten et al., 1992; Philiastides & Sajda, 2006).

A control analysis was performed on temporally aligned VEP component peak amplitudes. For each component, we selected the electrode with the highest activity in the individual average across conditions and jitter levels. Subsequently, amplitudes at that electrode were determined for each condition and jitter level at the individual peak latency. Peak amplitude was submitted to a multilevel linear regression with jitter as a covariate and Condition (Contour and Surface fixed) as a factor. This regression analysis followed a simplification strategy that proceeded with the evaluation of nested models in which a likelihood ratio test determined if the inclusion of a particular term significantly improved the fit (Kingdom & Prins, 2016; Knoblauch & Maloney, 2012).

### Multivariate pattern analysis

In order to test when task-relevant information about shapes appears in VEP signals, we applied single-trial Linear Discriminant Analysis (LDA) classification on stimulus orientation (vertical/horizontal), for Contour and Surface fixed conditions separately. The application of LDA EEG data has proved to be particularly powerful in predicting individual responses given the patterns of activation across electrodes (Grootswagers, Wardle, & Carlson, 2016).

LDA was at each time point between −100 and +500 ms from stimulus onset. No further pre-processing steps were taken other than those illustrated in the previous section. For each participant, the class distribution of vertical/horizontal labels was balanced by randomly removing trials from the majority class (He & Garcia, 2009). A common shrinkage factor of 0.007 was obtained by averaging individual estimates (oracle approximating shrinkage estimator – (Chen, Wiesel, Eldar, & Hero, 2010)). This regularization factor was then used in the estimation of the covariance matrix of EEG electrodes (Blankertz et al., 2011; Haufe et al., 2014). Finally, a 10-fold-cross validation scheme was applied to safeguard against overfitting (Grootswagers et al., 2016). Within-participant classification performances were submitted to a cluster-based permutation analysis to account for the temporal correlation structures (two-tailed test, 50,000 repetitions at a nominal *p* value of 0.05). LDA classification weights were projected onto the EEG electrodes to create scalp topographies of discrimination performance (Grootswagers et al., 2016).

## Results

### Behavioral session

**Figure 3.**
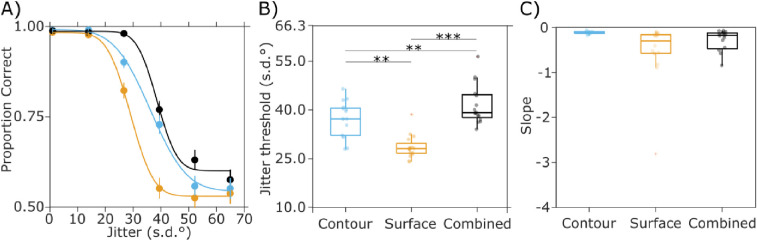
Results from the behavioral session. (A) shows the curves from probit regression for each experimental condition, data aggregated across participants. Error bars represent binomial 95% confidence interval (CI) for each estimated proportion of correct response. (B) Discrimination thresholds (inflection point of the curve) across participants, with significant differences between conditions indicated. (C) Slopes (gradients at inflection point) of the behavioral function per condition.

We estimated individual discrimination thresholds (inflection points) for the Surface, Contour, and Combined conditions and asked whether the simultaneous presence of surface and contour cues improved performance. A one-way repeated measures analysis of variance (ANOVA) with factor Condition (Contour, Surface, and Combined) yielded a significant Condition effect (F_(2,15)_ = 49.81, *p* < 0.01, and η^2^ = 0.54), see Figure 3. Post hoc *t*-test analysis, corrected for false discovery rate at *p* < 0.01 (Genovese et al., 2002), showed that discrimination thresholds were significantly higher for the Combined than the Contour alone (41.6 ± 3.2° vs. 37.1 ± 2.9°) and the Surface alone condition (28.6 ± 1.9°; *p* = 0.0036 and *p* < 0.001, respectively). This improved performance means that when both cues were present, participants could tolerate more jitter and still make correct judgments (about) 75% of the time. We also found that discrimination thresholds in the Contour condition were significantly higher than in the Surface condition (*p* < 0.01). No significant differences were found for the slopes (gradients at inflection point) across conditions. This indicates that the rate of change of discriminability across levels of jitter was statistically indistinguishable in the single and combined cue conditions.

### EEG session

#### Behavioral

**Figure 4.**
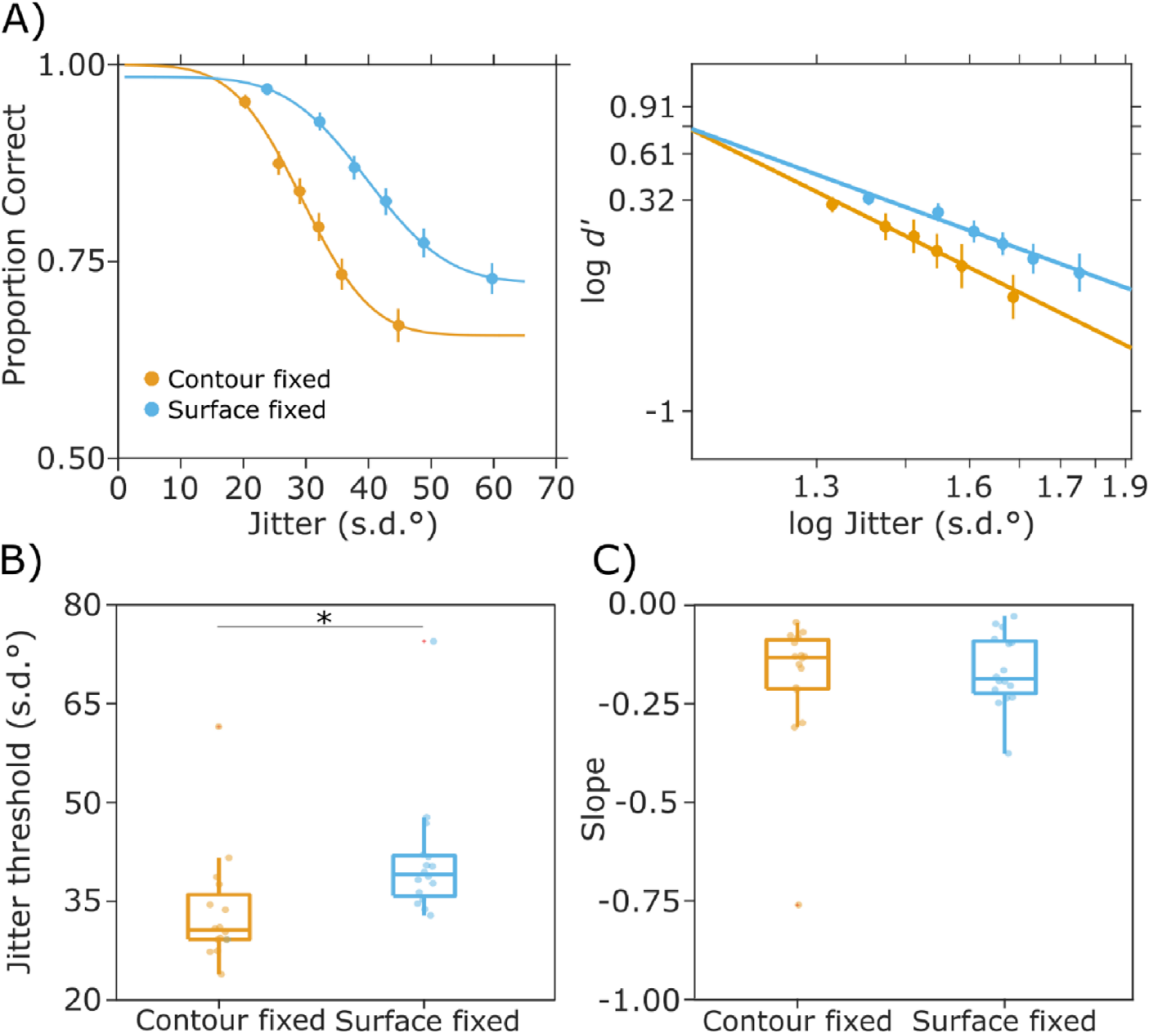
Behavioral results from the EEG session. (A) shows the fitted probit function in the two conditions for averaged data across participants (left panel), and the corresponding function based on d′ (right panel). Error bars represent 95% confidence interval (CI). (B and C) show the distribution of thresholds (inflection point of psychometric curve) and slopes (gradient at inflection point), respectively, with significant difference indicated.

During EEG recordings, participants discriminated combined stimuli while either the contour or surface cues were held at a fixed, individually calibrated jitter level. A one-way repeated measures ANOVA on the estimated discrimination thresholds (inflection points) nonetheless revealed a significant effect of condition (41.3 ± 4.7 vs. 33.47 ± 5.2; F_(1,15)_ = 7.93, *p* < 0.05, and η^2^ = 0.54) - see Figure 4. This means that despite the effort placed in equating the difficulty of the two conditions, the Surface fixed condition was easier. Between slope values (gradient at inflection point), no significant differences were found (−0.19 ± 0.09 vs. −0.17 ± 0.05; F_(1,15)_ = 0.18, *p* = n.s). The average reaction times were 822 ± 119 ms and 829 ± 85 ms for the Surface and Contour fixed conditions, respectively.

#### EEG

**Figure 5.**
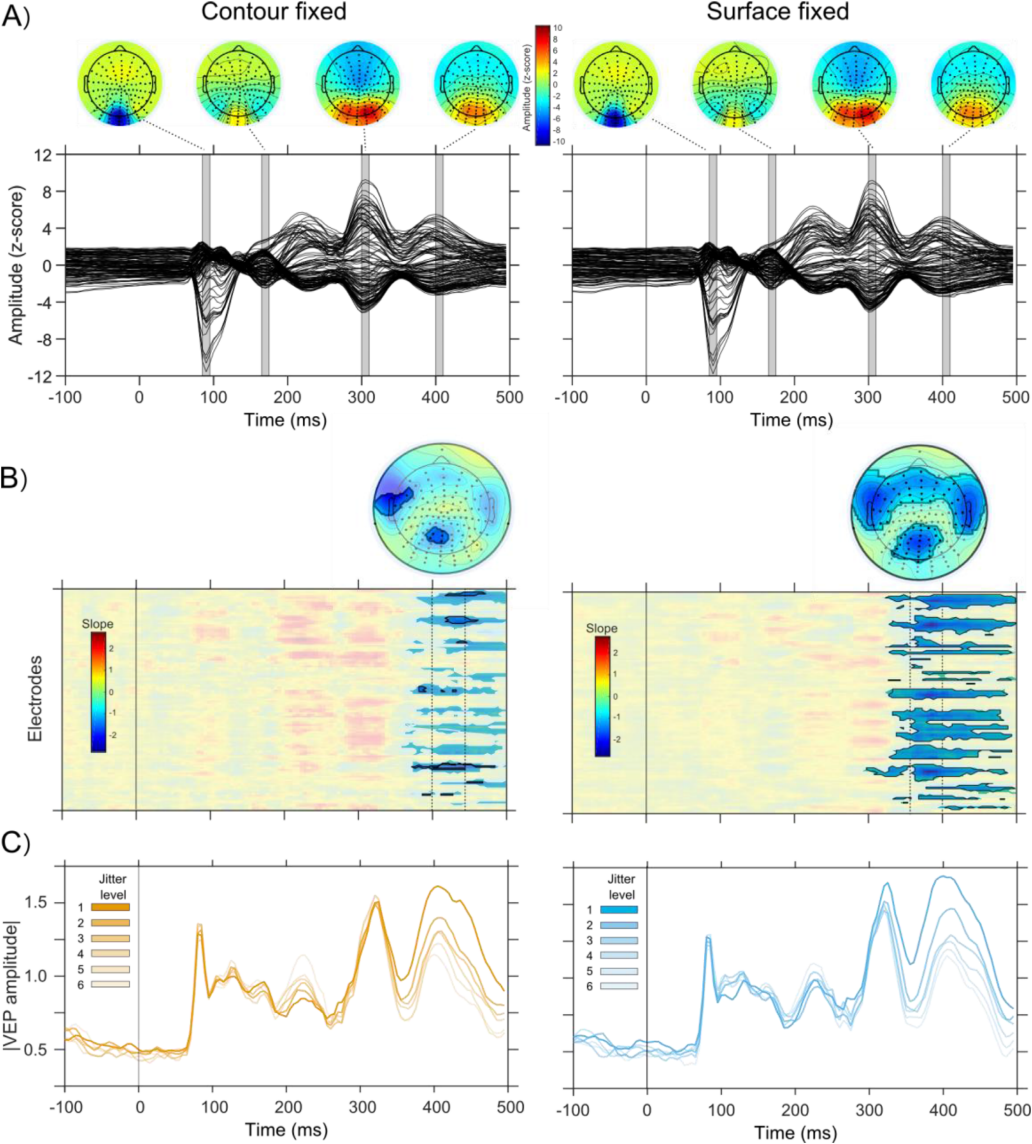
EEG results. (A) Grand-averaged VEPs of Contour and Surface fixed condition, respectively, with corresponding voltage topographies at the peak of four observed evoked components. (B) Shows slope estimates (in time for each electrode) of VEP-jitter regression in the two conditions. All statistically significant clusters (corrected for multiple comparisons) are highlighted. Within these clusters, black boundaries highlight regions where slope values that fell within the 95% confidence interval (CI) of the behavioral d′ slopes. The corresponding scalp distribution of slopes is shown on top (significant electrodes highlighted), in the interval delimited by the vertical dashed lines. (C) Grand-averaged traces across the central electrodes identified B, illustrates the linear decrease in amplitudes with increases in cue jitter for the two conditions.

To identify at what latencies and electrodes uncertainty was reflected in VEPs, we first linearly regressed recorded amplitudes against individual jitter values. For the Surface fixed condition, this analysis revealed significant clusters of non-zero slopes from around 300 ms after stimulus onset (Figure 5A). Each of these clusters were associated with negative slopes, meaning that VEP amplitudes decreased with jitter, and no positive slopes reached statistical significance. Within the clusters of statistically significant slopes, we then identified points with slopes that matched the ones observed in behavioral performance (the *d’-*jitter association). We found that these were located at electrodes over occipital, parietal, temporal, and frontal cortex.

Similar results were obtained in the Contour fixed condition, where significant clusters of non-zero slopes appeared from around 350 ms. Again, only negative significant slopes were observed and they were located at electrodes over occipital, parietal, and frontal cortex. A subset of electrodes over parietal and left temporal cortex showed slopes that fell within the 95% confidence interval of behavioral performance (see Figure 5B).

#### Response-locked analysis

We found that cue reliability is reflected around 400 ms after stimulus onset, beyond typical shape processing latencies. This is surprising given that for low jitter levels a shape is clearly visible, but for high jitter levels it is not, as evidenced by the behavioral results (see Figures 3 and 4). A possible explanation for this late effect is that the observed effects reflect decisional or response initiation processes that are more precisely time-locked to the behavioral response than to stimulus onset (Ales et al., 2013; Cottereau et al., 2014; Gevins et al., 1989; Kohler et al., 2018; Philiastides et al., 2014; Plomp et al., 2009). If this is the case, then the effects of reliability will be most pronounced when time-locking the VEPs to the behavioral response.

**Figure 6.**
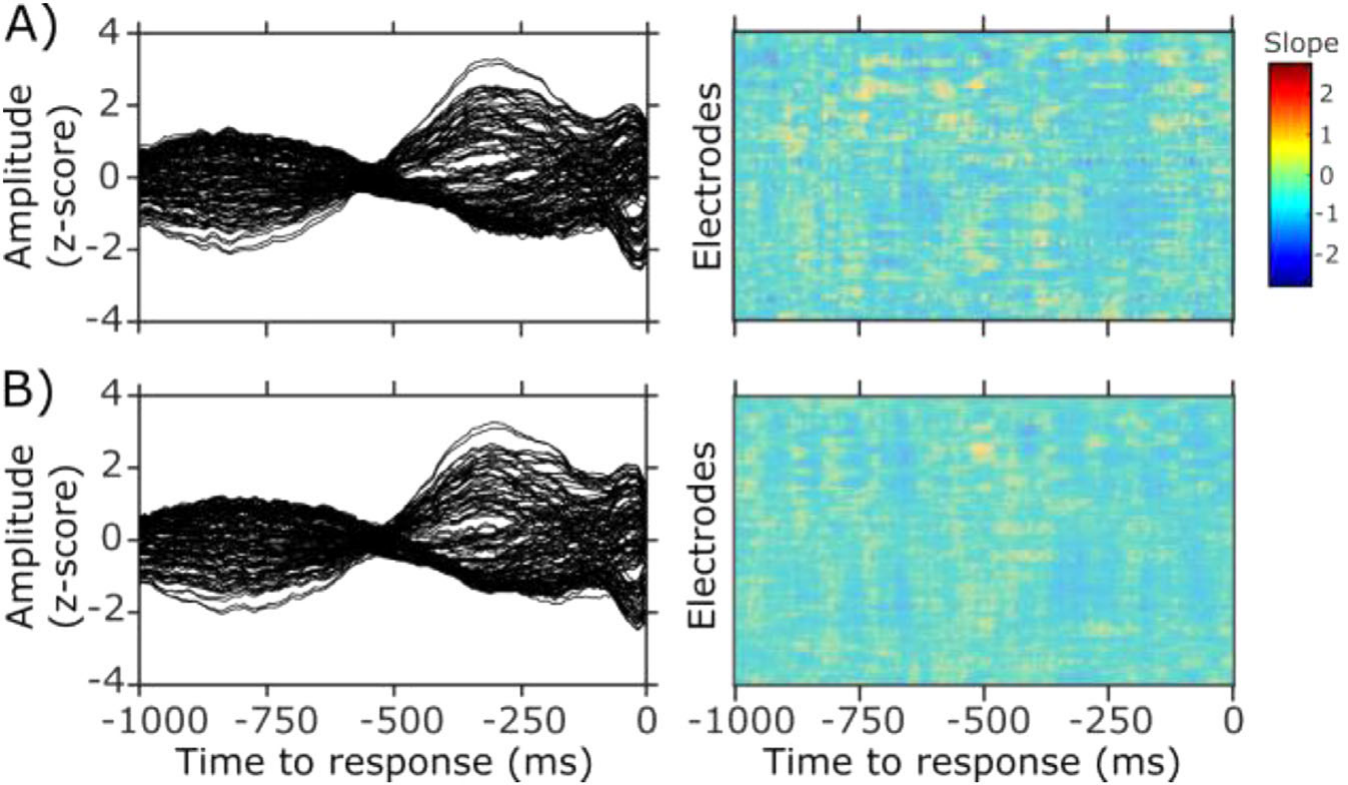
Response-locked results. (A and B) Show Contour and Surface fixed condition, respectively. The left plot presents the grand-averaged response-locked EEG activity, right plot the estimated slopes for all electrodes in time. No statistically significant clusters of non-zero slope were observed.

To investigate this, we repeated the regression analysis after aligning the EEG data to the button press. This response-locked analysis, however, did not identify any clusters with significant slopes in either Contour or Surface fixed condition (see Figure 6). This shows that cue reliability effects are not systematically time-locked to response initiation or execution processes.

#### Peak amplitude and latency analysis

The second possible explanation for the absence of effects at early latencies is that VEP components were not consistently time-locked to stimulus onset across participants (Spencer et al., 2000). We therefore investigated whether individual variability in the timing of evoked components obscured the systematic relations with jitter. Three visual evoked components could be reliably identified in all participants, with peak latencies at 89.9 ± 11.1, 307.2 ± 10.9, and 402.1 ± 27.9, respectively (see Figure 5). The N170 was only evident in half of the participants and, therefore, not analyzed further.

**Figure 7.**
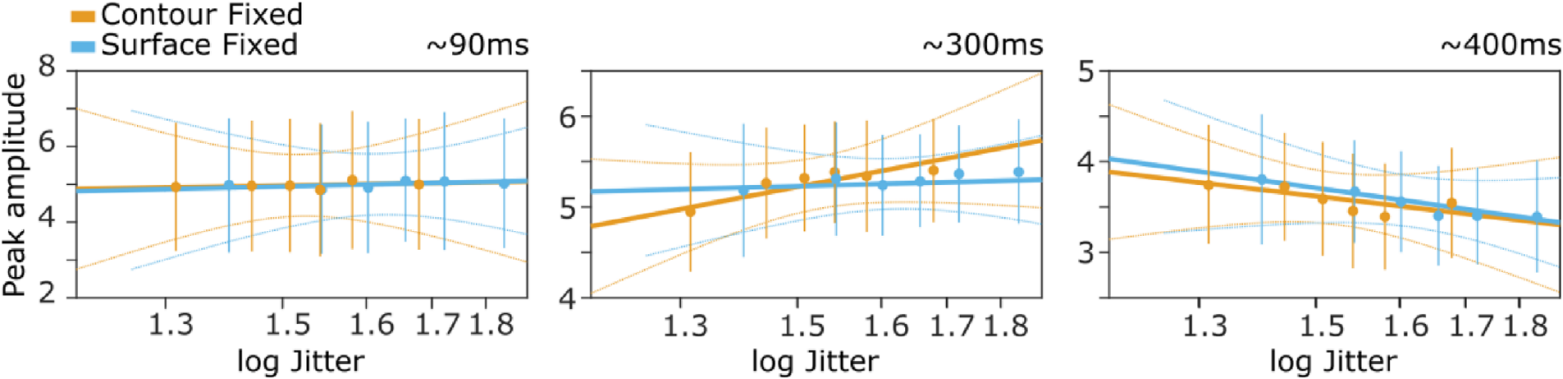
Temporally aligned peak amplitudes as a function of cue jitter. Error bars indicate 95% confidence intervals (CIs), thin lines reflect 95% CI of the estimated regression lines. The only negative association between VEP amplitude and cue jitter was found at a positive component peaking at around 400 ms after stimulus onset (P400).

Both the first component and the one around 300 ms revealed a statistically significant positive association between peak amplitude and jitter: the amplitude increased linearly with the log of jitter (0.55 ± 0.40, *p* < 0.01 and 1.46 ± 1.21, *p* < 0.05 for the first component and the one at 300 ms, respectively; Figure 7). At the 300 ms latency, this association was significantly less pronounced in the Surfaced fixed condition (as witnessed by the significant interaction: −1.26 ± 1, *p* < 0.05). In contrast, the peak amplitude at around 400 ms was negatively associated with the log of jitter (−1.02 ± 0.74, *p* < 0.01) in both Contour and Surface fixed condition. Together, the results from peak-aligned components confirm that negative associations between jitter and amplitude occur only at long latencies.

### Multivariate pattern analysis

**Figure 8.**
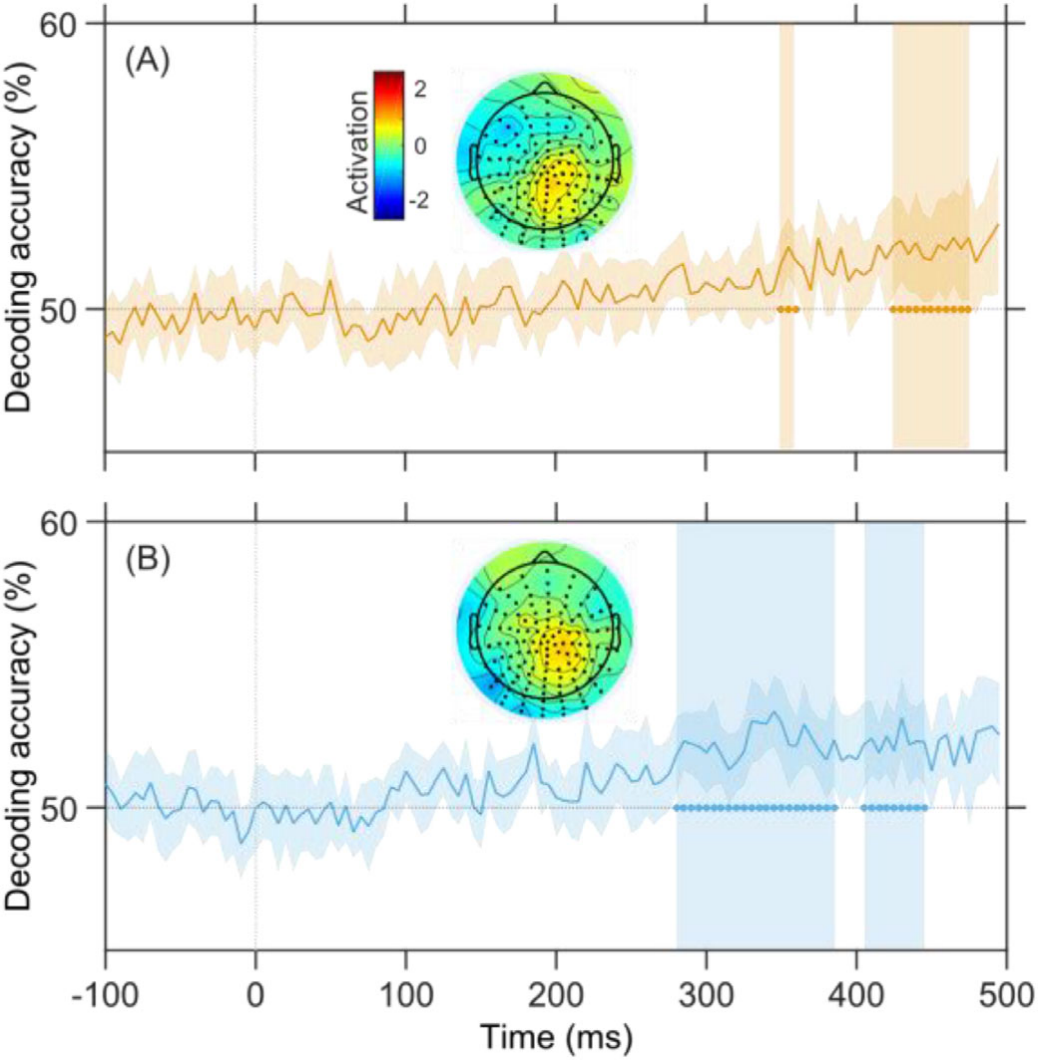
EEG decoding results. Top and bottom panels show results from the Contour and Surface fixed condition, respectively. Temporal evolution of the classification accuracy of the LDA along with a 95% confidence interval (CI) envelope. Cluster-based permutation identified significant temporal bins late in the trial evolution. The topographical maps portray the reconstructed signal activation based on estimated LDA weights, averaged across the vertical shaded areas.

As a last control analysis, we used an LDA decoder to determine at what latencies information about the ellipsoid shape is reflected in the VEPs. The results were in line with our previous findings: information about the ellipsoid discriminability appears quite late in the trial (about 300–350 ms for the Surface and Contour fixed, respectively), see Figure 8. Furthermore, we inspected which electrode drove the classification performance using a weight projection technique that reconstructs the neural activation patterns (Grootswagers et al., 2016; Haufe et al., 2014). The activation maps associated with peak discrimination accuracy suggested a prominent activation of central electrodes, at longer latencies (Surface fixed: 320–340 and 425–445 ms; Contour fixed: 350–360 and 425–445 ms). These results are consistent with our modelling results in that they suggest that relevant information about the ellipsoid shape is only present at late latencies, and over parietal areas.

## Discussion

We investigated how variations in cue reliability affect the spatiotemporal dynamics of visual evoked responses, using behavioral modeling to establish a principled link between brain states and sensitivity to cue reliability. We simultaneously presented contour and surface cues in a shape discrimination paradigm and found that VEPs parametrically reflect cue reliability at relatively long latencies, beyond 360 ms after stimulus onset, at electrodes over parietal and lateral frontal brain areas. Similar findings were independently obtained for the Contour fixed and Surface fixed conditions. In further analyses, we found that cue reliability effects were not time-locked to the behavioral response, and that they occurred at latencies when signals reflected stimulus orientation, as shown by an independent machine learning analysis.

We developed a model-based analysis pipeline that linked VEP amplitudes to behaviorally derived linear functions of *d’* to determine at what latencies evoked activity reflects individual discrimination capacity. First, participants’ rectified psychometric functions were derived by transforming probabilities of correct responses to discriminability values (*d’*) and regressing them against jitter to obtain slopes of behavioral discriminability. Separately, we regressed rectified VEP amplitudes against jitter levels and retained the data points where the VEP slopes matched the slopes of behavioral discriminability. This way we revealed latencies and electrodes where VEP amplitudes increase with cue reliability at the same rate that behavioral discriminability increases with reliability. Previous work studied how information about uncertain sensory evidence is neurally represented and by linking this representation to behavioral outcomes they suggested a unified psychophysical-neural model (Boyle et al., 2017; Philiastides & Sajda, 2006). The main advantage of our methodology lies in establishing an equivalence with the sensitivity to cue reliability (i.e. via the rectified *d’* function): the fit of behavioral and EEG data is essentially the same with the extra benefit of not having to impose arbitrary limits on VEP amplitudes based on behaviorally derived asymptotes. This methodology produced novel results that could be validated with an independent decoding analysis as explained below.

Our results revealed a surprising finding: we only observed effects of cue reliability at longer latencies, well after the typical latencies of visual processing that show contour, surface, and shape processing. Neural encoding of cue reliability may be expected to occur earlier in the evoked response because reliable cues form shapes and objects, whereas unreliable cues do not. Contour and surface grouping are considered mid-level vision mechanisms: collinear elements group into contours and shapes, and iso-oriented lines form surfaces that instantiate figure-ground segregation (Loffler, 2008; Wagemans et al., 2012). Such processes are thought to be elementary and are reflected at latencies before 200 ms with line and surface stimuli (Chicherov et al., 2014; Lamme, 1995; Scholte et al., 2008). Even more complex stimuli, such as objects and faces, evoked specific EEG signals between 100 and 200 ms (Bentin et al., 1996; Cichy et al., 2014; Isik et al., 2014; Itier & Taylor, 2004; Kirchner & Thorpe, 2006; Plomp et al., 2010). For example, in the context of a face/car discrimination task, there is evidence that the N1, at around 170 ms, can proportionally reflect stimulus visibility manipulations that resemble our variations in local cue reliability (Philiastides, 2006; Ratcliff et al., 2009). Our findings seem at odds with these observations and we therefore did follow-up analyses to determine what drove the late effects of cue reliability.

We excluded the possibility that individual variability may have hindered detecting effects of cue reliability at earlier visual evoked components. Time-locking the analysis to stimulus onset, and averaging across participants only preserves effects that are precisely temporally aligned across participants (Spencer et al., 2000). To account for possible inter-participant variability we temporally aligned visual evoked components that could be reliably identified in each participant. At around 400 ms, this analysis confirmed a clear negative association between VEP amplitudes and jitter (see Figure 7), reinforcing the idea of late effects of cue reliability. The analysis also confirmed that temporally corrected peak amplitudes at around 100 and 300 ms after stimulus onset did not show increased amplitudes with cue reliability. Rather, these components showed the opposite effect, with a linear increase of amplitudes for less reliable cues. This suggests that cue reliability is in some way reflected at earlier latencies, although these effects are not reliably time-locked to stimulus onset across participants in our data. One explanation for these positive slopes is that they reflect task difficulty (i.e. increased amplitudes for more difficult conditions; Philiastides et al., 2006; Philiastides et al., 2014). Alternatively, similar amplitude increases have previously been attributed to predictions about stimuli (Alink et al., 2010; Murray et al., 2002), but because we did not systematically vary surprise or prediction in this study, this interpretation remains speculative.

To independently determine at what latencies EEG signals contain information about the task-relevant shape, we applied LDA classification across jitter levels on the two response-categories (horizontal and vertical; Blankertz, Lemm, Treder, Haufe, & Müller, 2011; Grootswagers, Wardle, & Carlson, 2016; Haufe et al., 2014). EEG decoding can be expected to be more sensitive than electrode-by-electrode analysis because it capitalizes on single trials and simultaneously evaluates signals from all electrodes. Previous decoding analyses have shown that shape information can be decoded from early latencies onward (sometimes < 100 ms) (Cichy et al., 2014; Isik et al., 2014). In our data, however, decoding analysis showed similarly late findings as with the behavioral modeling approach. We found that shape discrimination signals become available at around 300 ms in the Surface fixed condition and after 400 ms in the Contour fixed condition. This decodability of signals into shapes at long latencies supports the notion of a late effect of stimulus reliability, as obtained with the behavioral modeling approach.

We thus obtained converging evidence from peak-amplitude, LDA, and model-based analyses that differences between shapes that can be reliably discriminated and ones that cannot be reliably discriminated, need not happen at the early latencies that are typically associated with visual processing. What could explain this absence of earlier effects in our results? It seems unlikely that the Gaborized stimuli alone account for the late effects. Our stimuli were designed to reflect segmentation of shapes from background in scenes, and they leveraged contour and surface grouping mechanisms using ellipsoid shapes embedded in noise (Hess et al., 2003; Loffler, 2008). With similar displays, contour and shape effects have been previously observed at around the N170 component (Bowden et al., 2015; Machilsen et al., 2011; Mathes et al., 2006; Shpaner et al., 2013), which reflects the processing of shapes, complex objects, and faces (Itier & Taylor, 2004; Philiastides & Sajda, 2006; Plomp et al., 2010). Shape detection in such displays results from grouping operations and complex recursive interplay among different brain regions that typically manifest before or around 300 ms after stimulus onset (Mijović et al., 2014; Scholte et al., 2008). Furthermore, we used a discrimination task that requires processing the shape as a whole, and we calibrated difficulty at individual sensitivity. Despite these stimulus characteristics and their task relevance, our stimuli did not reliably generate a distinctive N170 component, nor any shape effects before 300 ms after stimulus onset.

The observed late effects of cue reliability may reflect a cue combination process. In our stimuli, task-relevant shapes were formed by two simultaneously presented cues - the aligned contour and surface elements. In this way, our stimuli reflect natural scenes better than when only one cue is presented. In the behavioral session, we observed improved performance when both cues were available compared with when only one cue was presented (see Figure 3), a cue combination effect in line with previous work (Machilsen & Wagemans, 2011). In the EEG session, one cue was held constant at individual, performance-derived levels while the other cue was varied. Because both cues were task-relevant, participants are expected to use their combined information for perceptual decisions (Landy et al., 1995). Given that low-level differences were carefully controlled for in our displays, and that we did not find typical VEP signatures of contour and surface processing, we interpret the late cue reliability effects to reflect the integration of surface and contour cues under varying levels of cue reliability. In other words, contour and surface cues are combined and the variations in their reliability are resolved at a relatively late stage of processing.

Late VEP components are thought to reflect target detection, evidence accumulation for perceptual decisions, as well as motor planning or execution (Heekeren et al., 2008; Philiastides et al., 2014; Plomp et al., 2009). In perceptual decisions, prior to committing to a choice, stimulus evidence is evaluated and integrated over time - in that sense, the late representation of stimulus reliability may reflect the ongoing accumulation of sensory evidence in decisional units. Electrodes over parietal areas have been shown to reflect evidence accumulation in perceptual decisions. In a face-car discrimination task, VEPs at long latencies (> 300 ms) reflected the amount of randomization of the images (i.e. the reliability of local cues), and were well-modeled as an evidence accumulation process (Philiastides et al., 2014; Philiastides & Sajda, 2006). However, the effects of cue reliability in our data differ from evidence accumulation processes in important ways. The scalp topography associated with evidence accumulation includes parietal electrodes but also frontal electrodes associated with motor command (Donner et al., 2009; Gold & Shadlen, 2007; Polanía et al., 2014; Wyart et al., 2012), resembling a P300 topography, defined spatially by one centrally located and circularly shaped positivity (Linden, 2005; Picton, 1992; Twomey et al., 2015). In our data, however, the topography that reflected cue reliability did not resemble a classical P300 topography. In our VEP data, the distribution of positive amplitudes over posterior areas around 300 ms after stimulus onset seems more indicative of ongoing visual processing than of target detection and decision processes. In addition, the traces from electrodes that showed cue reliability effects do not reflect a drift-diffusion trajectory of linear increases with time (see Figure 5C; c.f. Philiastides et al. 2014, see Figure 2). Another indication that cue-reliability effects are different from evidence accumulation is that the effects are not tightly linked to response processes. We found that ERPs time-locked to the button press showed no systematic effects of cue reliability (see Figure 6), whereas previous work showed that time-locking the analysis to the response preserves evidence accumulation processes (Philiastides et al., 2014), and can unveil decision-related information in visual areas (Ales et al., 2013; Cottereau et al., 2014; Kohler et al., 2018). Our response-locked results likewise refute the idea that pre-motor processes play a role. Taken together, these considerations suggest that our results do not reflect decision or motor outputs per se, but rather that cue reliability affects VEPs before participants commit to a choice - as if unreliable cues impaired the ability to form and maintain stable decisions prior to the behavioral outputs (Kubovy & Healy, 1977).

Our findings, therefore, most likely reflect late, distributed perceptual processes. Previous work has shown that P400 amplitudes decrease when stimuli are ambiguous in their interpretation (Kornmeier et al., 2016). This effect held for a variety of high-level visual stimuli and was interpreted as an evaluation of the reliability of perceptual processing results. Likewise, our results may be best interpreted as an evaluation of the reliability of visual inputs, as a late decision-related process that precedes the behavioral choice. This notion is strengthened by the following considerations. Despite the attempt to equate the difficulty of the Surface and Contour fixed conditions, we found a superior behavioral performance in the case of the Surface fixed condition (contour reliability varied). Increased sensitivity for contour over surface information is supported by animal recordings where neural responses to surface stimuli decrease with the distance to boundary signals (Lee, Mumford, Romero, & Lamme, 1998). This difference can be leveraged to diagnose at which stage, in the architecture of visual processing, our results are located. The first cluster of significant associations between VEP and jitter in the Contour fixed condition appeared before 400 ms, whereas in the Surface fixed condition they appeared after 400 ms (see Figure 5B). This observation was corroborated by the decoding analysis in which discriminant signals appeared at shorter latencies in the Surface fixed (prior 300 ms) than in the Contour fixed (after 300 ms) condition (see Figure 8). The result suggests that perceptual processes are slower when the system is engaged in the resolution of more difficult stimuli such as our Contour fixed stimuli. The recurrent interplay between brain areas involved in the grouping of elements requires greater efforts for displays mainly defined by surface cues; consequently, the effect of cue reliability is delayed as well.

Although several control analyses confirmed our conclusion, the absence of earlier cue reliability effects can also be due to methodological limitations. First, our statistical analysis used appropriate corrections for multiple testing (Bullmore et al., 1999; Maris & Oostenveld, 2007), but this may have led to the exclusion of weaker and less reliable effects from the results. An example of this is the early, reversed cue reliability effect that became statistically significant after temporal peak alignment (see Figure 7). Second, recorded EEG potentials reflect the sum of activity (ion currents) throughout the brain, but EEG is not uniformly sensitive to currents in all 3D directions (Nunez & Srinivasan, 2006). The EEG picture of the underlying brain activity is therefore imperfect and allows for the possibility, in principle, that cue reliability effects occurred at earlier latencies but escaped detection, due to unfavorable orientations or because of cancelation of weak currents. Although we can therefore not conclude with certainty that cue reliability is reflected only at long latencies, our data do indicate that the late effects are stronger and more reliable. Complementary work using fMRI, magnetoencephalography (MEG), or intracranial recordings could help to further establish whether cue reliability effects exist in visual areas, at earlier latencies (Foucher et al., 2003; Hermes et al., 2017; Itthipuripat et al., 2019).

In conclusion, our results show a late VEP component that reflects cue reliability under conditions where separate cues are integrated. This implies that mid-level visual displays do not necessarily invoke robust mid-level sensory processes, but can involve late decision-related activity that reflects the reliability of the presented local cues. At these latencies, the underlying brain activity involves a complex mixture of activity and interactions (Mijović et al., 2014; Plomp et al., 2015; Scholte et al., 2008), the exact nature of the underlying sources, their interactions, and how this effectively evaluates cue reliability remains to be further investigated.
